# Granulocyte abundance and maturation state at diagnosis predicts treatment-free remission in CML

**DOI:** 10.1038/s41375-025-02769-2

**Published:** 2025-09-16

**Authors:** Mikko Purhonen, Mikael Tatun, Katariina Luukkainen, Kevin Hung, Henri Sundquist, Oda Tafjord, Stina Söderlund, Shady Adnan-Awad, Anni Dohlen, Johanna Heikkinen, Perttu Koskenvesa, Sanna Siitonen, Satu Mustjoki, Naranie Shanmuganathan, Coral Bryce, Signe Danielsson, Henrik Hjorth-Hansen, Ulla Olsson-Strömberg, Takashi Kumagai, Shinya Kimura, David M. Ross, Oscar Brück

**Affiliations:** 1 Hematoscope Lab, Comprehensive Cancer Center, Helsinki University Hospital, Helsinki, Finland; 2 Department of Oncology, University of Helsinki, Helsinki, Finland; 3 Department of Clinical Chemistry, HUS Diagnostic Center, University of Helsinki and Helsinki University Hospital, Helsinki, Finland; 4 Royal Adelaide Hospital and SA Pathology, Central Adelaide Local Health Network, Adelaide, SA Australia; 5 Department of Hematology, St. Olavs Hospital, Trondheim, Norway; 6 Department of Medical Science and Division of Hematology, Uppsala University Hospital, Uppsala, Sweden; 7 Hematology Research Unit Helsinki, Department of Hematology, University of Helsinki and Helsinki University Hospital Comprehensive Cancer Center, Helsinki, Finland; 8 ICAN Digital Precision Cancer Medicine Flagship, University of Helsinki and Helsinki University Hospital Comprehensive Cancer Center, Helsinki, Finland; 9 Translational Immunology Research program, University of Helsinki, Helsinki, Finland; 10 Department of Hematology, Helsinki University Hospital Comprehensive Cancer Center, Helsinki, Finland; 11 Department of Clinical Chemistry, University of Helsinki and Helsinki University Hospital, Helsinki, Finland; 12 Precision Medicine Theme, South Australian Health and Medical Research Institute, Adelaide, SA Australia; 13 Medicinska kliniken, Universitetssjukhuset, Orebro, Sweden; 14 Department of Cancer Research and Molecular Medicine, Norwegian University of Science and Technology (NTNU), Trondheim, Norway; 15 Department of Hematology, Ome Medical Center, Ome-Shi, Tokyo, Japan; 16 Division of Hematology, Respiratory Medicine and Oncology, Department of Internal Medicine, Faculty of Medicine, Saga University, Saga, Japan; 17 Department of Haematology, Flinders University and Medical Centre, Adelaide, SA Australia

**Keywords:** Chronic myeloid leukaemia, Translational research, Chronic myeloid leukaemia

## Abstract

Treatment-free remission (TFR) has become a therapeutic objective for selected chronic-phase chronic myeloid leukemia (CP CML). However, no standardized biomarker is yet in clinical use. In this multi-center study, we explored the potential of bone marrow (BM) cytomorphology given its global accessibility and integral role in clinical diagnostics. We included diagnostic BM aspirate samples of 113 CP CML patients from seven clinical sites having attempted first TKI discontinuation. We digitized cytomorphological slides into 100x-magnified high-resolution images and analyzed these with deep learning-based image analysis. We profiled the BM cytomorphological fingerprint of CP CML patients and recapitulated the known granulocytic predominance and reduction of lymphoid, monocytic and erythroid cells in comparison to an extensive cohort of 942 control BM samples. We discovered neutrophil abundance and granulocytic maturation to associate with sustained TFR. We confirmed these visually and demonstrated their independent impact over known clinical factors. Our study underlines the potential of computational BM cytomorphology to identify novel clinical biomarkers and suggests that granulocytic expansion and maturation at diagnosis could reflect intrinsic disease pathology influencing TFR maintenance.

## Introduction

Chronic myeloid leukemia (CML) is a hematological malignancy characterized by the *BCR::ABL1* fusion gene, producing a dysregulated tyrosine kinase enzyme and leading to uncontrolled production of myeloid cells in the bone marrow (BM) [1]. Most CML patients are diagnosed at chronic phase (CP).

The use of tyrosine kinase inhibitors (TKIs) targeting the *BCR::ABL1* kinase [2] has transformed CML into a manageable disease, with patients having a life expectancy comparable to that of the healthy population [3]. Several TKI discontinuation trials have demonstrated that approximately 50% of patients can sustain molecular response (MR) five years after discontinuation, consolidating treatment-free remission (TFR) as a novel goal of therapy [4–7]. To this day, several biomarkers have been suggested to predict TFR success, illustrating the complexity of mechanisms regulating TFR. These include the total duration of TKI treatment [8–11], duration of deep molecular response [9, 12–14], and activity of natural killer cells at the time of treatment discontinuation [15, 16]. In addition, several studies have demonstrated an association between early *BCR::ABL1* kinetics and outcome [17–21]. Despite these promising findings, no biomarker can robustly identify patients best suited for a discontinuation attempt.

BM aspiration slides are routinely assessed at the diagnosis of CP CML patients. These represent a globally accessible and affordable resource to evaluate the BM cellular composition, maturation, and morphological landscape. Recent advances in digital pathology and modern computational methods have made it possible to study BM morphology beyond diagnostics [22, 23]. In this multi-center study, we explore the BM cytomorphology of CP CML patients with deep learning-based image analysis. We investigated diagnostic samples to focus on the expanded myeloid compartment, giving us insights into the underlying disease biology within each patient. The primary objective of this study was to identify novel biomarkers of TFR as well their clinical correlates. Collectively, our work highlights the underexplored potential of BM cytomorphology to better understand disease trajectories of CML and possibly other blood cancer patients.

## Methods

### Patients

We included in total 113 adult CP CML patients having attempted first TKI discontinuation in routine practice or in clinical trials from seven clinical sites: Royal Adelaide Hospital (Adelaide, Australia, *n* = 60), Saga University Hospital (Saga, Japan, *n* = 12), Ome Medical Center (Tokyo, Japan, *n* = 11), St. Olavs Hospital (Trondheim, Norway, *n* = 8), Uppsala University Hospital (Uppsala, Sweden, *n* = 8), Helsinki University Hospital (Helsinki, Finland, *n* = 8), Medicinska kliniken (Örebro, Sweden, *n* = 6). No patient died during the follow-up period or restarted the TKI treatment before relapse.

We collected two reference cohorts from Helsinki University Hospital. The first cohort (*n* = 13) consisted of diagnostic BM samples from CP CML patients who failed to response to TKI treatment and did not try treatment discontinuation. Failure was defined according to European LeukemiaNet 2020 criteria at 3, 6, and/or 12 months [2]. The second cohort (*n* = 942) consisted of BM samples from individuals without active hematological disease and with comparable age (44-64 years) and sex distribution to the main cohort. Samples were collected for reasons including diagnostic procedures performed alongside allogeneic stem cell donation, persistent abnormal blood counts without subsequent hematological diagnoses, or follow-up of patients in long-term molecular remission. A study permit was approved by the Helsinki University Hospital Medical Research Ethics committee (HUS/1077/2025) and institutional research boards at each clinical site before the start of the study. Informed consent was obtained from all participants prior to their involvement in the study. The study adhered to the Declaration of Helsinki.

### Data collection

Investigators or associated biobanks collected data from their respective clinical sites. Data included MGG-stained BM aspirate slide and information about patient and treatment characteristics at diagnosis:

1. Patient age and sex.
2. Laboratory values: peripheral blood (PB) white blood cell and platelet counts (E9/L). PB differentials (%) of blasts, basophils, eosinophils and lymphocytes. BM blast differentials (%). Spleen size measured as the distance below the costal margin, determined either by palpation or imaging.
3. The EUTOS long-term survival (ELTS) score, categorical risk classes, and respective variables.
4. Treatments: name and generation of TKIs across treatment lines.
5. Treatment response: *BCR::ABL1* transcript levels at 3, 6, and 12 months. MR4.0 achievement and duration prior to TFR.
6. Transcript types: e14a2 and e13a2.

In addition, we included BM differentials defined by morphologists from 56 samples from Adelaide, Australia to visually validate computationally derived differentials.

### Slide digitization

Slides were scanned at two magnification levels using West Medica’s HemaVision Ultimate digital microscope. First, we scanned the whole slide image (WSI) at 10x magnification. Then, we selected technically representative regions of interest (ROIs) and scanned these at 100x magnification with oil immersion. The study included both squash and wedge-type BM slides. In squash slides, ROIs were selected from areas near connective tissue islets while avoiding regions that were technically suboptimal, overcrowded with cells, or containing a high concentration of disrupted cells. In wedge slides, we selected comparable ROIs from the distal section of the slide.

### Image analysis of BM cytomorphology with the Cellbytes application

**Fig. 1:**
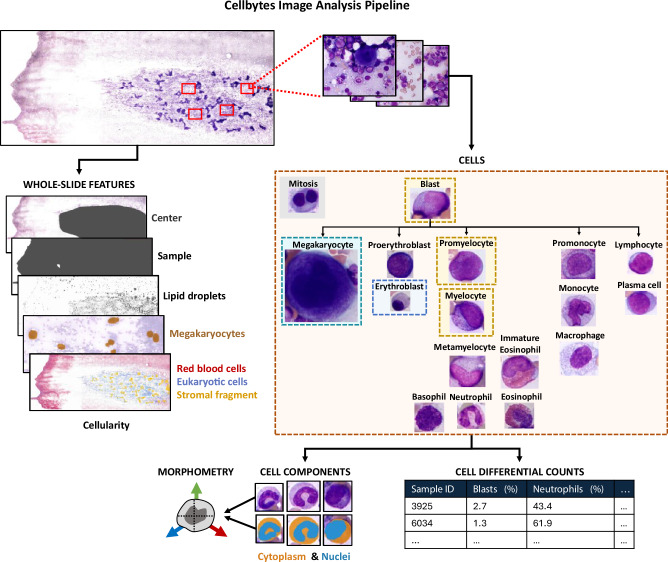
Cellbytes image analysis pipeline. Dataflow and outputs of the Cellbytes image analysis, shown separately for whole-slide image (WSI) and region-of-interest (ROI) image analyses.

We analyzed the BM slides with the Cellbytes application, an in vitro diagnostic medical software (Cellbytes Ltd., Helsinki, Finland) intended for assessment of BM aspirate slide images. The application provides image analysis algorithms, analyzing the WSIs at 10x and ROIs at 100x magnification (Fig. 1, Supplementary Fig. 1). The algorithms form a full cytomorphological evaluation of the sample, cell composition, and occurrence of dysplasia in megakaryocytes, erythroblasts, or granulocytic precursors comparable to clinical assessment performed by pathologists. In addition, the pipeline extracts experimental information on cellular morphometry.

The algorithms are categorized into computer vision and downstream algorithms (Supplementary Fig. 1). Computer vision algorithms make predictions directly from images with deep learning methods, e.g., detecting and classifying cells. Downstream algorithms analyze processed cytomorphological data generated by the computer vision algorithms. Computer vision algorithms utilize various Convolutional Neural Network (CNN) architectures [24, 25], as well as the Vision Transformer (ViT) [26] architecture. Downstream algorithms utilize machine learning methods to predict sample-level properties, e.g., sample cellularity. Statistical metrics are utilized to analyze the morphometry of single cells.

The software uses a ViT-based foundation model as a backbone for all 100x-level computer vision algorithms. Instead of using a general base model, such as ImageNet-pretrained model, the backbone was trained to learn meaningful cell features specifically occurring in cytomorphological samples. The backbone utilizes self-supervised learning (SSL) methodology based on the Masked Siamese Networks framework [27]. The training dataset included 27 million unlabeled, single-cell images from multiple clinical sites and digital scanners. Cell classification accuracy was validated on random samples from all centers by an experienced hematopathologist.

### Preprocessing of cytomorphology data

Due to variations in technical quality of the slides, identification and exclusion of low-quality 100x image areas were crucial for reliable analysis of the cells. We applied two preprocessing strategies (Supplementary Methods). First, we excluded low-quality 100x images and cells before analyzing cell differentials. Next, we conducted low-quality cell exclusion based on cell morphometry, before analyzing cell-level morphology. In the first preprocessing, we removed 7 samples not meeting the criteria for 300 intact cells. The final analysis included 106 samples with a median of 2202 intact cells (IQR, 1605–2626). After the second preprocessing, the samples had a median of 1499 intact cells (IQR, 893–1898).

### Statistical analysis

Patient characteristics were summarized for the study cohort (Table 1). For continuous variables, we used the Wilcoxon rank-sum test to compare two groups. The Chi-squared test was used for categorical variables. Spearman’s rank correlation coefficient was used for measuring correlation between continuous variables. We used Kaplan-Meier curves to visualize time-to-event data and determined the optimal cut-off values using the maximally selected log-rank statistic, with *P*-values adjusted using Conditional Monte Carlo method.

**Table 1:** Patient characteristics of the study cohort and association of prognostic variables with TFR maintenance at 36 months (interval of 33–39 months) after TKI discontinuation.

Variable	All patients in the study cohort	Number of patiens missing the value	Patients in maintained TFR at 36 months	Patients relapsed within 36 months	*P*-value
Sex^a^					1
Female	45 (42.5%)		23 (43.4%)	22 (41.5%)	
Male	61 (57.5%)		30 (56.6%)	31 (58.5%)	
Patient age at diagnosis (years)^b^	53 [44–64]		51 [43–63]	55 [47–65]	0.12
ELTS risk class^a^		4 (3.8%)			0.59
Low	76 (74.5%)		35 (70.0%)	41 (78.8%)	
Intermediate	21 (20.6%)		12 (24.0%)	9 (17.3%)	
High	5 (4.9%)		3 (6.0%)	2 (3.8%)	
*BCR::ABL1* Transcript type^a^		33 (31.1%)			
e14a2	39 (53.4%)		22 (57.9%)	17 (48.6%)	0.57
e13a2	35 (47.9%)		18 (47.4%)	17 (48.6%)	1
e14a2 and e13a2	12 (11.3%)		6 (11.3%)	6 (11.3%)	1
First-line TKI^a^					0.1
Imatinib	64 (60.4%)		32 (60.4%)	32 (60.4%)	
Nilotinib	19 (17.9%)		13 (24.5%)	6 (11.3%)	
Dasatinib	23 (21.7%)		8 (15.1%)	15 (28.3%)	
First-line TKI generation^a^					1
Imatinib	64 (60.4%)		32 (60.4%)	32 (60.4%)	
2G-TKI	42 (39.6%)		21 (39.6%)	21 (39.6%)	
Last TKI^a^					0.14
Imatinib	48 (45.3%)		23 (43.4%)	25 (47.2%)	
Nilotinib	22 (20.8%)		15 (28.3%)	7 (13.2%)	
Dasatinib	35 (33.0%)		14 (26.4%)	21 (39.6%)	
Bosutinib	1 (0.9%)		1 (1.9%)	0 (0.0%)	
Last TKI generation^a^					0.84
Imatinib	48 (45.3%)		23 (43.4%)	25 (47.2%)	
2G-TKI	58 (54.7%)		30 (56.6%)	28 (52.8%)	
*BCR::ABL1* transcript level^b^					
3 months (%)	0.32 [0.02–1.25]	27 (25.5%)	0.3 [0.01–1.18]	0.43 [0.05–1.61]	0.28
6 months (%)	0.04 [0.0–0.13]	26 (24.5%)	0.03 [0.0-0.08]	0.06 [0.01–0.2]	0.08
12 months (%)	0.01 [0.0–0.05]	32 (30.2%)	0.003 [0.0–0.024]	0.01 [0.002–0.065]	0.05
Time to MR4.0 (months)^b^	12.0 [6.03–27.02]	1 (0.9%)	12.0 [6.03–31.52]	12.0 [7.0–26.0]	0.75
MR4.0 at 12 months^a^		1 (0.9%)			1
Yes	57 (54.3%)		28 (53.8%)	29 (54.7%)	
No	48 (45.7%)		24 (46.2%)	24 (45.3%)	
Duration of MR4.0 (months)^b^	42.0 [34.0–64.0]	9 (8.5%)	47.7 [36.6–70.8]	40.0 [32.7–57.5]	0.09
Duration of treatment (months)^b^	58.5 [46.2–100.5]	1 (0.9%)	67.0 [48.0–117.3]	55.1 [43.0–75.0]	0.07

*TFR* Treatment-free remission, *TKI* Tyrosine kinase inhibitor, *MR4.0* Molecular response 4.0 (*BCR::ABL1* ≤ 0.01% on the International Scale; 4-log reduction from standardized baseline).

^a^N (%), Chi-squared test.

^b^Median [25–75%], Wilcoxon rank-sum test.

**Fig. 2:**
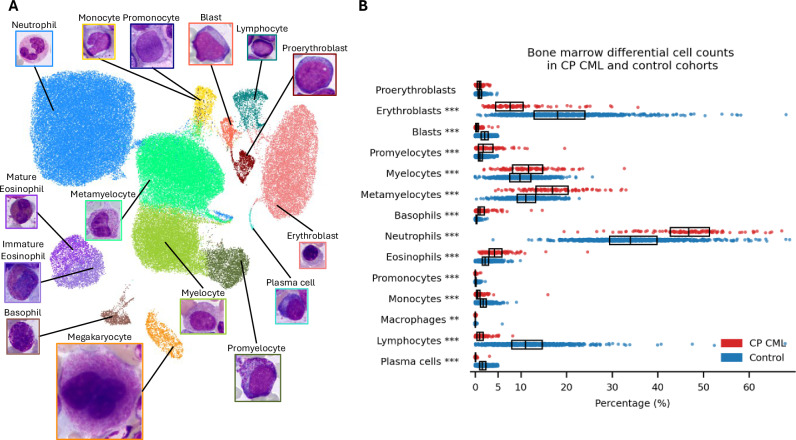
Cytomorphological characteristics of the study cohort. **A** Uniform manifold approximation and projection (UMAP) of the detected bone marrow (BM) cells based on cell classification confidences and morphology features. **B** BM cell differentials of the chronic phase chronic myeloid leukemia (CP CML) cohort at diagnosis (red) and control reference cohort (blue). The distribution of each cell type is visualized as a scatter plot and a box plot, showing the median and interquartile range (IQR). Wilcoxon rank-sum test was used to compare the distributions of CP CML and control samples. Asterisks indicate significance levels: *P < 0.05, **P < 0.01, ***P < 0.001.

We used uniform manifold approximation and projection (UMAP) to visualize individual cells in a 2D plane and assess the validity of the cell classification results (Fig. 2A). The projection was based on the predicted confidence values from the cell classification model and morphometry data.

All statistical analyses and visualizations were conducted with Python and R in the data secure HUS Acamedic environment.

## Results

### Clinical characteristics of the study cohort

Patient characteristics of the study cohort are reported in Table 1. We assessed TFR at 36 months after TKI discontinuation with molecular monitoring performed at 33–39 months. Patients who relapsed before or during this time window were classified as relapsing. The rate of relapse was 41.5% at 6 months after treatment discontinuation and 50.0% within 36 months aligning with previous studies. The criteria for TFR varied across centers, with MMR used for 75.5% and MR4.0 for 24.5% of patients relapsed within 36 months. However, all MR4.0 relapses occurred within 6 months, suggesting a rapid increase in *BCR::ABL1* transcripts and likely MMR relapse.

The cohort included a slightly higher proportion of male patients in line with the known sex ratio of CML. The median age at diagnosis was 53 years. Most patients were classified into either the low or intermediate ELTS risk groups, reflecting the inclusion criteria of TKI discontinuation studies. Imatinib was the most commonly used first-line treatment (60.4%), while the second generation (2G) TKIs were slightly more common as last-line treatments (54.7%). Nilotinib was the most common 2G-TKI in Australia, whereas in other centers dasatinib was more commonly prescribed. In addition, one patient received bosutinib as a last-line treatment. The median duration of TKI treatment before discontinuation was 4.9 years. Except for one patient, who did not achieve MR4.0, the median duration of sustained MR4.0 prior to discontinuation was 42 months.

### Granulocytes dominate the BM cell composition of CP CML patients

We studied the cell composition and maturation of CP CML patients using cytomorphological BM aspirate samples from 7 clinical sites and a deep learning-based image analysis software (Fig. 1). UMAP visualization of individual cells demonstrated robust clustering by cell type (Fig. 2A). Cells are grouped according to their lineage and maturation stage as shown by the continuum from promyelocytes towards myelocytes, metamyelocytes and neutrophils. Similar transitions are visible for the erythroid and eosinophil lineages. Mature granulocytes are clustered into neutrophils, eosinophils, and basophils, and the maturation stage of eosinophils can be distinguished.

When examining more closely, the BM cell differentials followed a distribution anticipated for CP CML patients, differing from controls (Fig. 2B). Compared to controls, we observed an increase in the granulocytic lineage (median 86.8% vs. 60.5%), covering both in the immature (median 32.2% vs. 22.2%) and mature (median 53.2% vs. 36.6%) compartments. While neutrophils represented the predominant cell type in both CML and controls, their proportion was significantly higher in CP CML patients (median 46.7% vs. 34.0%). The expansion of the granulocyte compartment in CML bone marrow extended to metamyelocytes, myelocytes, eosinophils, promyelocytes, and basophils, in descending order of abundance.

In consequence, the proportion of the lymphoid and monocytic compartments was reduced in CP CML patients compared to control subjects (Fig. 2B). The blast proportion remained low, meeting the criteria for CP CML. We also observed a substantial erythroid subpopulation and large variation in their number, but in median, the proportion of erythroid cells was significantly lower compared to controls (median 8.6% vs. 19.0%).

### Granulocyte maturation is associated with TFR maintenance

**Fig. 3:**
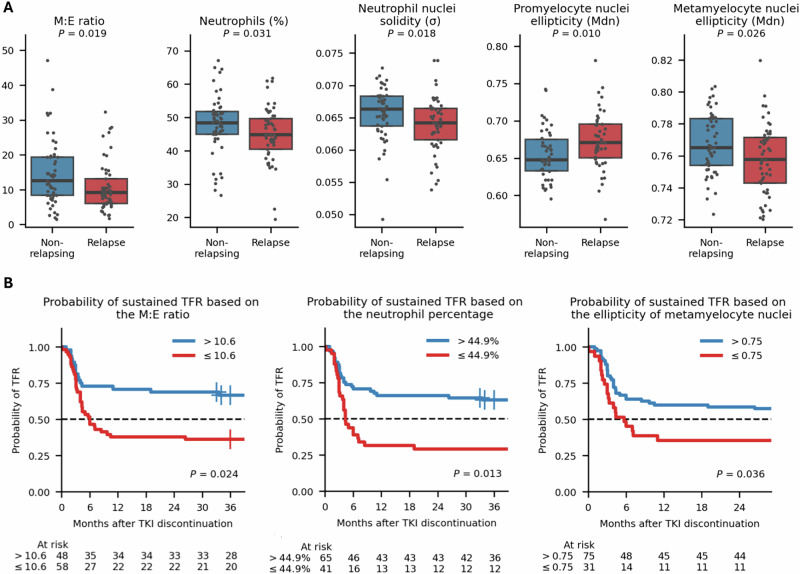
Association of cytomorphological variables with treatment-free remission (TFR) maintenance at 36 months after TKI discontinuation. **A** Box plots with individual data points of the most significant cytomorphological variables, categorized by TFR maintenance, with *P* values shown (Wilcoxon rank-sum test). **B** Kaplan–Meier plots showing the probability of TFR according to the cytomorphological variables. Risk groups were defined for each variable based on the optimal cut-off value maximizing log-rank statistics: blue lines represent lower risk of relapse, and red lines indicate higher risk. Statistical comparisons were performed using the log-rank test, with adjusted *P*-values shown. A dashed horizontal line indicates the 50% probability of TFR. Abbreviations: σ Standard deviation, Mdn Median. M:E Ratio, Myeloid to erythroid ratio where myeloid cells included promyelocytes, myelocytes, metamyelocytes, neutrophils, eosinophils, basophils, promonocytes, and monocytes. Erythroid cells included proerythroblasts and erythroblasts.

To study the association of BM cytomorphology with TFR status, we first compared non-relapsing patients with patients relapsed within 36 months and subsequently divided the relapsed group into early and late relapsing patients. We identified five cytomorphology variables that were significantly associated with TFR (Fig. 3A). Two of these described granulocyte proportions and three of their morphological properties. Non-relapsing patients had a higher myeloid to erythroid (M:E) ratio (median 12.6 vs. 9.2). Non-relapsing patients also had an increased proportion of neutrophils (48.4% vs. 44.9%). We divided the patients into two groups according to the optimal cut-off value and compared their Kaplan-Meier estimates (Fig. 3B). The probability of TFR at 12 months was 66.2% in the group with elevated neutrophils (> 44.9%), compared to 31.7% in the group with decreased neutrophils. Similarly, patients with a high M:E ratio (>10.6) more often remained in remission (70.8% vs. 37.9%).

Given the robustness of the M:E ratio as a simple TFR biomarker and its application both for cytomorphological and histopathological examination, we aimed next to validate this visually. We compared the computationally calculated BM cell differential counts with the cell differentials defined by morphologists (Adelaide, Australia). As a standard practice, morphologists counted 500 intact cells, whereas the computational pipeline analyzed a median of 1907 intact cells (IQR 1401–2278). Both the erythroid cells (*R* = 0.75, *P* < 0.001) and the M:E ratio demonstrate strong correlation (*R* = 0.78, *P* < 0.001; Supplementary Fig. 2). In samples characterized with a large M:E ratio, humans tended to report higher values than the algorithm.

**Fig. 4:**
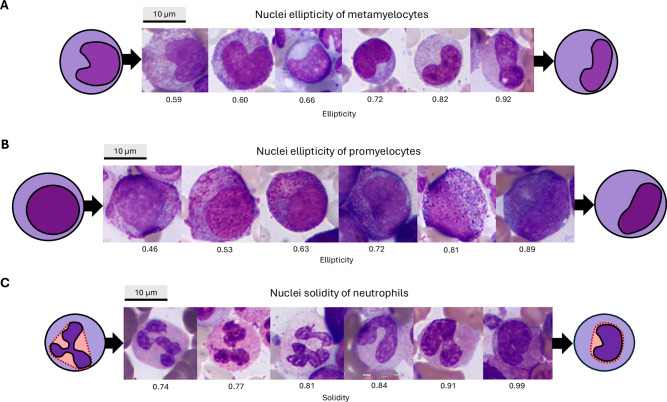
Representative cell images with varying morphometry. **A** Metamyelocytes with varying ellipticity values of nuclei. Ellipticity is defined as one minus the ratio of an object’s minor axis to its major axis, where 0 represents a perfect circle and 1 an infinitely stretched object. **B** Promyelocytes with varying ellipticity values of nuclei. **C** Neutrophils with varying solidity values of nuclei. Solidity measures the ratio of an object’s area to its convex area. Lower values reflect greater nuclear contour complexity, such as hypersegmentation.

Next, we examined morphological properties linked with TFR. We observed that metamyelocytes of non-relapsing patients had more elliptic nuclei compared to relapsed patients (Figs. 3A and 4A). Kaplan–Meier analysis revealed that patients with increased metamyelocyte nuclei ellipticity (> 0.75) had a higher probability of TFR (60.0% vs. 35.5%; Fig. 3B). In contrast, we observed that promyelocytes of non-relapsing patients had rounder nuclei compared to relapsed patients sharing more elliptical nuclei (Figs. 3A and 4B). However, we did not observe a statistically significant difference in the Kaplan-Meier estimates between the two groups, categorized based on the cut-off value of 0.66 (*P* = 0.112). In addition, values were available for 87.7% (93/106) of the samples, as some samples were excluded due to too few promyelocytes (Supplementary Methods). To visually illustrate these findings at the patient level, we gathered metamyelocyte (Supplementary Fig. 3) and promyelocyte (Supplementary Fig. 4) images with varying nuclei ellipticity values from one non-relapsing and one relapsed patient.

In addition, we observed that non-relapsing patients had more variation in neutrophil nuclear solidity (Fig. 3A). Solidity is a measure of how compact a shape is. It is calculated by dividing the area of the nucleus by the area of its convex hull, the smallest shape without indentations that can fully enclose the nucleus. Lower solidity values correspond to irregular or lobulated nuclei, which may indicate hypersegmentation, whereas higher values denote maturing band cells (Fig. 4C). This is aligned with the observation that an increased variation in solidity was associated with a lower median nuclei solidity in neutrophils (Supplementary Fig. 5A, B). Similarly to metamyelocytes and promyelocytes, we gathered neutrophil images from one non-relapsing and one relapsed patient (Supplementary Fig. 5C–E).

### BM cytomorphology at diagnosis differs by relapse rate

To better understand how BM cytomorphology is associated with relapse rate, we examined three patient groups: non-relapsing, late relapsed, and early relapsed. We divided the relapsed patients into early and late relapse groups using a 6-month threshold, based on the findings that majority of relapses occur within this period [5]. We also conducted additional comparisons with CP CML patients who failed to response to TKI treatment.

**Fig. 5:**
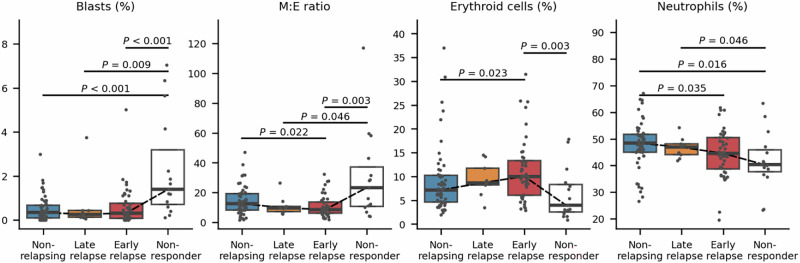
Comparison of cytomorphology between chronic myeloid leukemia (CML) patients. The analysis included four CML patient groups: patients maintaining treatment-free remission (TFR) at 36 months, patients with a late (> 6, ≤ 36 months) or early relapse (≤ 6 months) following tyrosine kinase inhibitor (TKI) discontinuation and patients failing to respond to TKI during the first year after diagnosis according to European LeukemiaNet 2020 criteria. Box plots with individual data points for each cytomorphological variable, categorized by patient groups, with *P*-values shown (Wilcoxon rank-sum test). The black dashed line connects the groups by their medians. Abbreviations: σ Standard deviation, Mdn Median. M:E Ratio, Myeloid to erythroid ratio where myeloid cells included promyelocytes, myelocytes, metamyelocytes, neutrophils, eosinophils, basophils, promonocytes, and monocytes. Erythroid cells included proerythroblasts and erythroblasts.

We observed a decreasing trend in the M:E ratio from non-relapsing to late relapse and early relapse (Fig. 5). However, in the group of non-responders M:E ratios were significantly higher, similar to blast proportion. A reversed trend was observed in the erythroid cell proportion. In contrast, we observed a decreasing trend in neutrophil proportion from non-relapsing to early relapsed and finally to the non-responder group. A decreasing trend was observed in the variation of nuclei solidity of neutrophils, but only non-relapsing and early relapse groups differed statistically (Supplementary Fig. 6). We did not observe distinct trends in promyelocyte and metamyelocyte nuclei ellipticity (Supplementary Fig. 6).

### High M:E ratio is associated with splenomegaly but not established TFR biomarkers

**Fig. 6:**
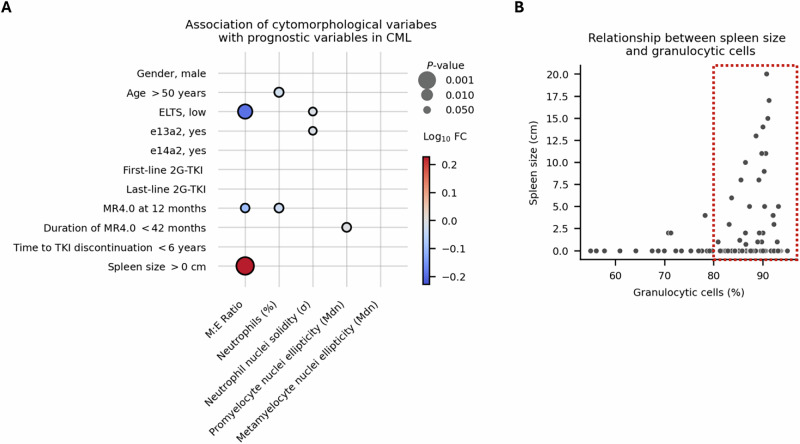
Association of cytomorphology and prognostic variables in chronic myeloid leukemia (CML). **A** Association of cytomorphological variables with clinical prognostic variables, assessed using the Wilcoxon rank-sum test. Balloon size indicates the strength of the association between the cytomorphological variable categorized by median value (*x*-axis) and the prognostic variable (y-axis). Red indicates a positive association, meaning that an increase in the value of cytomorphological variable is linked to the prognostic variable in the *y*-axis. In contrast, blue indicates a negative association. The intensity of the color reflects the magnitude of the difference, measured as log10-transformed fold-change. **B** Scatter plot showing the relationship between spleen size and the proportion of bone marrow (BM) granulocytic cells. Abbreviations: 2G Second generation, σ Standard deviation, Mdn Median. M:E Ratio, Myeloid to erythroid ratio where myeloid cells included promyelocytes, myelocytes, metamyelocytes, neutrophils, eosinophils, basophils, promonocytes, and monocytes. Erythroid cells included proerythroblasts and erythroblasts.

We studied the relationship between prognostic clinical and TFR-related cytomorphological variables (Fig. 6A). We observed a lower M:E ratio in ELTS low-risk patients, which was likely explained by the association between the increased M:E ratio and spleen enlargement (Supplementary Fig. 7). In fact, the association of ELTS risk score and spleen size with M:E ratio was mainly driven by the proportion of granulocytes. We observed that the majority of patients having an enlarged spleen, the proportion of BM granulocytes was over 80% (Fig. 6B). These observations are likely explained by increased extramedullary hematopoiesis or sequestration of excess granulocytes into the spleen [28]. The cytomorphological variables did not directly associate with the established prognostic variables, suggesting that these could represent independent biomarkers of TFR.

In addition, we studied the association between 3-month halving time and the most predictive cytomorphological variables in 52 patients from Australia and Finland with available data. We divided the patients into two groups based on the optimal cut-off value for halving time (12.0 days), which corresponded to the median value of the cohort. A shorter halving time increased the probability of TFR at 12 months (69.2% vs. 42.3%; Supplementary Fig. 8), but the difference was not statistically significant (*P* = 0.224). We did not observe any association between halving time and the cell types linked with TFR.

For comparison, we studied the association of common and established prognostic variables with TFR (Table 1). We did not observe statistical association between TFR and age, sex, ELTS, TKI type, TKI duration, or transcript type. Non-relapsing patients had lower *BCR::ABL1* transcript levels at 12 months (0.003 vs. 0.01, *P* = 0.05) after diagnosis. However, values were available only for 69.8% (74/106) of patients. Collectively, our findings suggest that BM cytomorphology could be integrated to refine and optimize the currently applied clinical variables.

### Erythroid and granulocyte abundance is linked with higher maturation rate

Cytomorphology was associated with TFR through variations in cell proportions and morphology. However, variations in cell proportions might reflect morphological differences not directly associated with TFR. In contrast, morphological variations might relate to variations in cell proportions. We aimed to understand these connections better and studied the association between the proportion and morphology in cell types linked with TFR.

Given that the M:E ratio emerged as a promising biomarker of TFR maintenance, we examined first how the morphology of precursor erythroid cells was associated with their morphology. Erythroblasts demonstrated greater nuclear compactness and cell solidity, and a smaller cytoplasmic area in samples with erythroid abundance (Supplementary Fig. 9). When examining closer, we observed that these samples contained smaller, smoothly shaped cells with round nuclei possibly reflecting higher proportion of orthochromatic erythroblasts.

Next, we studied the association between nuclei ellipticity and cell proportions in both promyelocytes and metamyelocytes. We observed that higher promyelocyte nuclei ellipticity was associated with elevated promyelocytes (2.9% vs. 1.6%, *P* = 0.008). In contrast, lower metamyelocyte nuclei ellipticity was associated with elevated metamyelocytes (17.9% vs. 15.2%, *P* = 0.008; Supplementary Fig. 10A).

Lastly, we examined neutrophil cytomorphology but could not observe any association between these and neutrophil proportion. However, we observed that neutrophil nuclei solidity was associated with the proportion of metamyelocytes (Supplementary Fig. 10B–D). Lower nuclear solidity, i.e., increased hypersegmentation, was associated with decreased metamyelocytes.

## Discussion

Here, we studied the role of BM cytomorphology at the time of diagnosis in predicting TFR maintenance in CP CML patients at 36 months after the treatment discontinuation. The underlying biology of TFR remains poorly understood, and no standardized biomarker has been established for routine clinical use. In this study, we found that an increased proportion and maturation of granulocytes at diagnosis are associated with sustained TFR.

We explored the BM fingerprint of CP CML patients, e.g., the cellular composition, maturation, and morphological changes of distinct cell lineages, using a novel deep learning-based software designed for clinical use. To validate its performance in CP CML patients, we mapped the BM cell composition, recapitulating the known pre-dominance of neutrophils and maturing granulocytes in comparison to controls. We demonstrated that simple tools, such as the M:E ratio associated with TFR. A recent study demonstrated that a higher neutrophil count in PB at the time of treatment discontinuation is associated with sustained TFR in CP CML [29]. The rate of granulocytic expansion could be associated with disease-specific factors such as genomic alterations in myeloid driver genes, which have been shown to occur in 26% of CP CML patients and to associate with TFR [30]. Factors that affect hematopoietic lineage commitment and differentiation might be reflected in the M:E ratio [31, 32]. In addition, antitumor neutrophils have been identified as a significant component of the cancer microenvironment [33]. However, these factors remain speculative. Granulocytic expansion could simply reflect disease burden or prolonged disease before diagnosis. Features linked to disease burden or chronicity, such as high clinical risk score [34], blast count [35], and the presence of additional genomic variants [30] are typically associated with a reduced probability of TFR.

In addition to overproduction of granulocytes, we found evidence that patients maintaining TFR showed a predominance of mature over immature granulocytes. Non-relapsing patients were characterized with neutrophils that demonstrated more variation in nuclear segmentation and shift towards nuclear hypersegmentation, indicating more mature neutrophils. In contrast, relapsed patients harbored more promyelocytes with elliptic nuclei and metamyelocytes with rounder nuclei. Cytomorphological examination of promyelocytes did not demonstrate clear signs of maturation based on the cell size or cytoplasm. However, increased ellipticity may indicate a shift toward myelocytes with more banded nuclei.

We also found that morphological changes were associated with cell proportions. Higher promyelocyte nuclei ellipticity was associated with elevated promyelocytes and could reflect a shift in proliferation or maturation. Neutrophil and metamyelocyte morphology were associated with metamyelocyte proportion. This could be explained with the left shift phenomenon where maturing metamyelocytes and band neutrophils become enriched in clinical conditions associated with reactive neutrophilia. Analogical to this, round-shaped nuclei in metamyelocytes could reflect similar phenomenon of metamyelocytes from myelocytes.

These findings may be absent from single-cell transcriptomic and immunophenotypic studies, which often focus on selected cell types. Density-based separation methods isolate mononuclear in the upper interface layer, while denser granulocytes fall to the bottom and are typically excluded. In contrast, morphological assessment of BM slides allows the evaluation of the full granulocyte spectrum.

Categorization of patients into non-relapsing, late, and early relapsed patients showed differences in the proportion and the maturation of erythroid and granulocytic cells. BM neutrophil proportion indicated both higher risk for relapse after TKI discontinuation and failure to respond to TKI treatment.

To our knowledge, similar deep learning-based image analysis has not been used to study CML. BM examination is a routine procedure in the diagnosis of myeloid malignancies, but relies on human vision, which has limitations. First, humans cannot efficiently process large data volumes. Typically, routine examinations cover no more than 500 cells from an aspirate sample containing millions of cells. Second, visual examination is subjective, leading to interobserver variability. This is particularly evident in CML, where differentiating cells and detecting subtle morphological changes can be challenging due to high cell counts. The presented software addresses these limitations by analyzing thousands of cells per sample and utilizing deep learning models trained on a multicenter dataset using SSL, known to increase model generalization [36]. The framework is designed to mimic the decision-making process of a hematopathologist and to provide novel, detailed insights into BM morphology.

While the methodology has notable strengths, we recognize its limitations. Slide appearance varied between centers due to variations in sample preparation (squash vs. wedge), staining, and processing techniques. To mitigate the effects of technical variability, we selected ROIs manually to analyze the most representative regions, and an experienced hematopathologist validated cell classification results. We also excluded technically suboptimal samples and cells, which might have affected cell distribution.

We also acknowledge that the analysis was conducted on diagnostic samples and that treatment decisions may have influenced the probability of TFR. In addition, TFR criteria varied across centers between MMR and MR4.0, but all MR4.0 relapses occurred within 6 months, suggesting a rapid increase in *BCR::ABL1* transcripts and likely MMR relapse. In line, a previous study has shown that the time to MMR and MR4.0 loss does not differ significantly, with only a 1-month difference in median when relapses occur within 6 months [37]. This difference may have introduced minor bias in time-dependent analyses. Lastly, we acknowledge that our study was a proof-of-concept, given the moderate sample size of 106 patients, and a larger study is needed to validate the clinical utility of these findings.

In conclusion, our study demonstrated the potential of the overlooked BM cytomorphology in identifying novel clinical biomarkers when analyzed with modern computational techniques. We suggest that granulocytic expansion and maturation at diagnosis could reflect essential disease pathology and inform treatment discontinuation decisions in CP CML patients, possibly improving quality of life, cost-effectiveness, and long-term disease management [9, 38, 39].

## Supplementary information

Supplementary Materials: Granulocyte Abundance and Maturation State at Diagnosis Predicts Treatment-Free Remission in CML

Supplementary Figures

## Data Availability

Due to the multi-site nature of the dataset, a study permit is required at each participating site. Please contact the corresponding author for data access.

## Code availability

Codes are available at https://github.com/purz1/cml_tfr.
