# Supplementary material for: Granulocyte abundance and maturation state at diagnosis predicts treatment-free remission in CML
Source: Leukemia. 2025 Sep 16;39(12):2968–77. doi: 10.1038/s41375-025-02769-2 (PMC12634435; doi:10.1038/s41375-025-02769-2)
Supplement: Supplementary file 1 — Supplementary Materials: Granulocyte Abundance and Maturation State at Diagnosis Predicts Treatment-Free Remission in CML [file 41375_2025_2769_MOESM1_ESM.docx]

**Supplementary Materials: Granulocyte Abundance and Maturation State at Diagnosis Predicts Treatment-Free Remission in CML**

**SUPPLEMENTARY METHODS**

***Preprocessing of cytomorphology data***

Exclusion of low-quality 100x images and cells:

1. Remove 100x images with over 40% artefacts to exclude technically suboptimal areas of the slide.
2. Remove any remaining low-quality erythroblasts, including technically pyknotic cells that were misclassified as erythroblasts.
3. Remove cells located at the image border.
4. Exclude samples with fewer than 300 intact cells.

Cell morphometry-based cell exclusion:

1. Remove low-quality granulocytes. A separate classification model is used to assess the quality of myelocytes, metamyelocytes, basophils, neutrophils, and eosinophils. Low quality indicates that the cell is shrunk or compressed by neighboring cells, which can lead to unreliable morphometry results.
2. Remove technically unrepresentative cells using classification models trained on cell-level morphometry variables.
3. Remove outlier cells based on morphometry feature values. We pooled the feature values from all samples for each cell class and filtered outlier values based on the distribution. If the distribution followed an approximately symmetric Gaussian distribution, cells outside the 1^st^ and 99^th^ percentiles were removed. If the distribution was skewed, cells outside the 90^th^ percentile on the tail were removed.
4. For each sample, exclude the cell morphometry variable if the cell class has fewer than 10 occurrences after filtering to avoid false discoveries based on too few cells.
