# Supplementary material for: Granulocyte abundance and maturation state at diagnosis predicts treatment-free remission in CML
Source: Leukemia. 2025 Sep 16;39(12):2968–77. doi: 10.1038/s41375-025-02769-2 (PMC12634435; doi:10.1038/s41375-025-02769-2)
Supplement: Supplementary file 2 — Supplementary Figures [file 41375_2025_2769_MOESM2_ESM.pdf]

SUPPLEMENTARY FIGURES

A

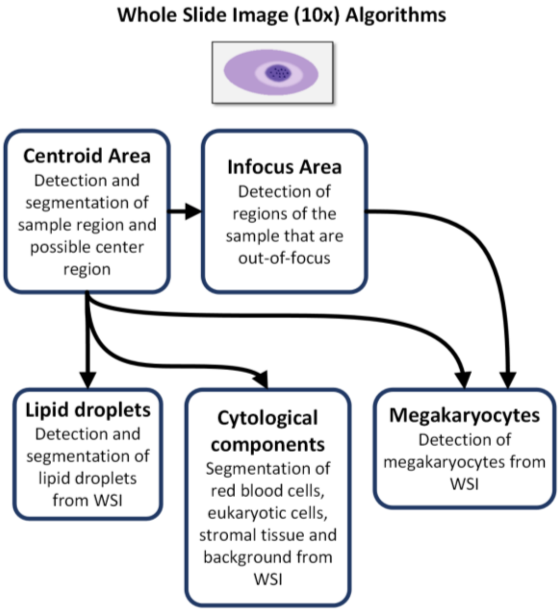

B

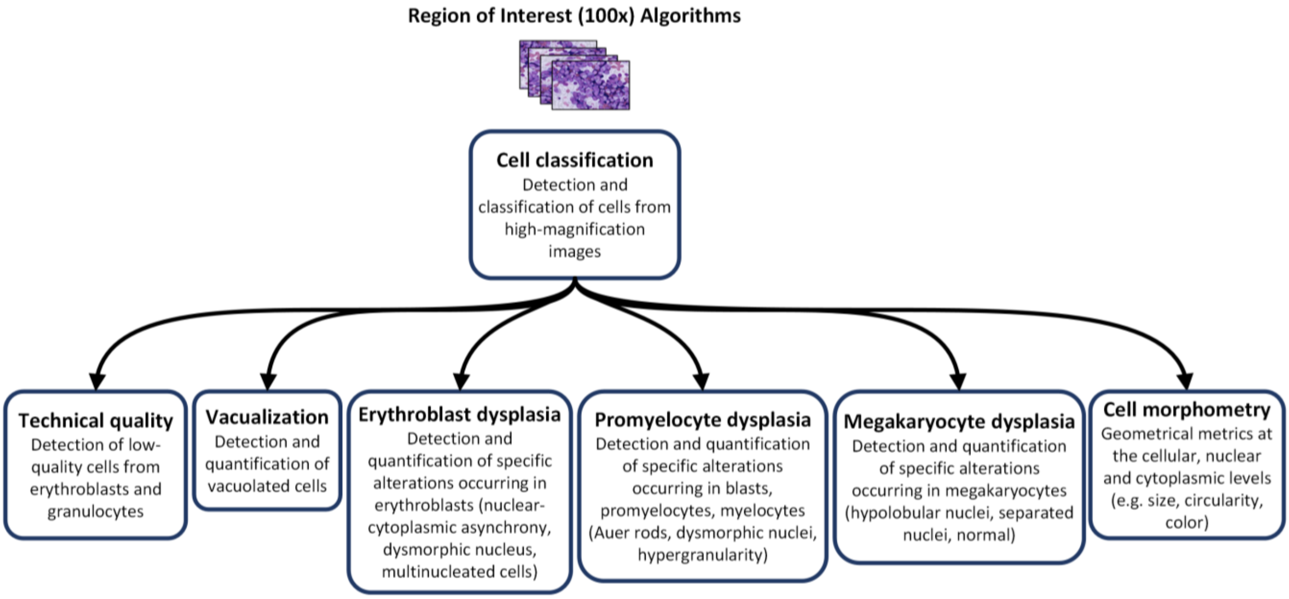

Supplementary Figure 1. Cellbytes image analysis algorithms. (A) Whole-slide image (WSI) algorithms. (B) Region of interest (100x) algorithms.

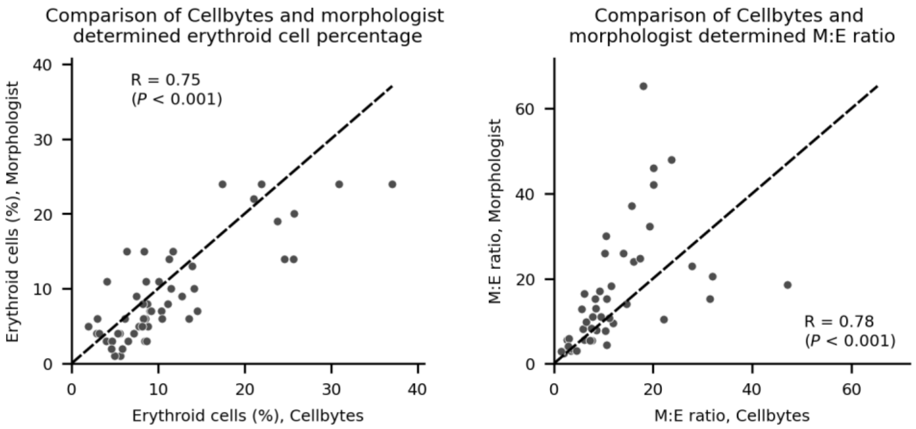

Supplementary Figure 2. Comparison of bone marrow (BM) cell differential counts of Cellbytes cell detector and a skilled cytomorphologist. Scatter plots for erythroid cells and M:E ratio, with optimal trend line. Spearman's rank correlation was used to compare the variables (correlation coefficient (R) and *P*-value shown). Abbreviations: M:E Ratio, Myeloid to erythroid ratio where myeloid cells included promyelocytes, myelocytes, metamyelocytes, neutrophils, eosinophils, basophils, promonocytes, and monocytes. Erythroid cells included proerythroblasts and erythroblasts.

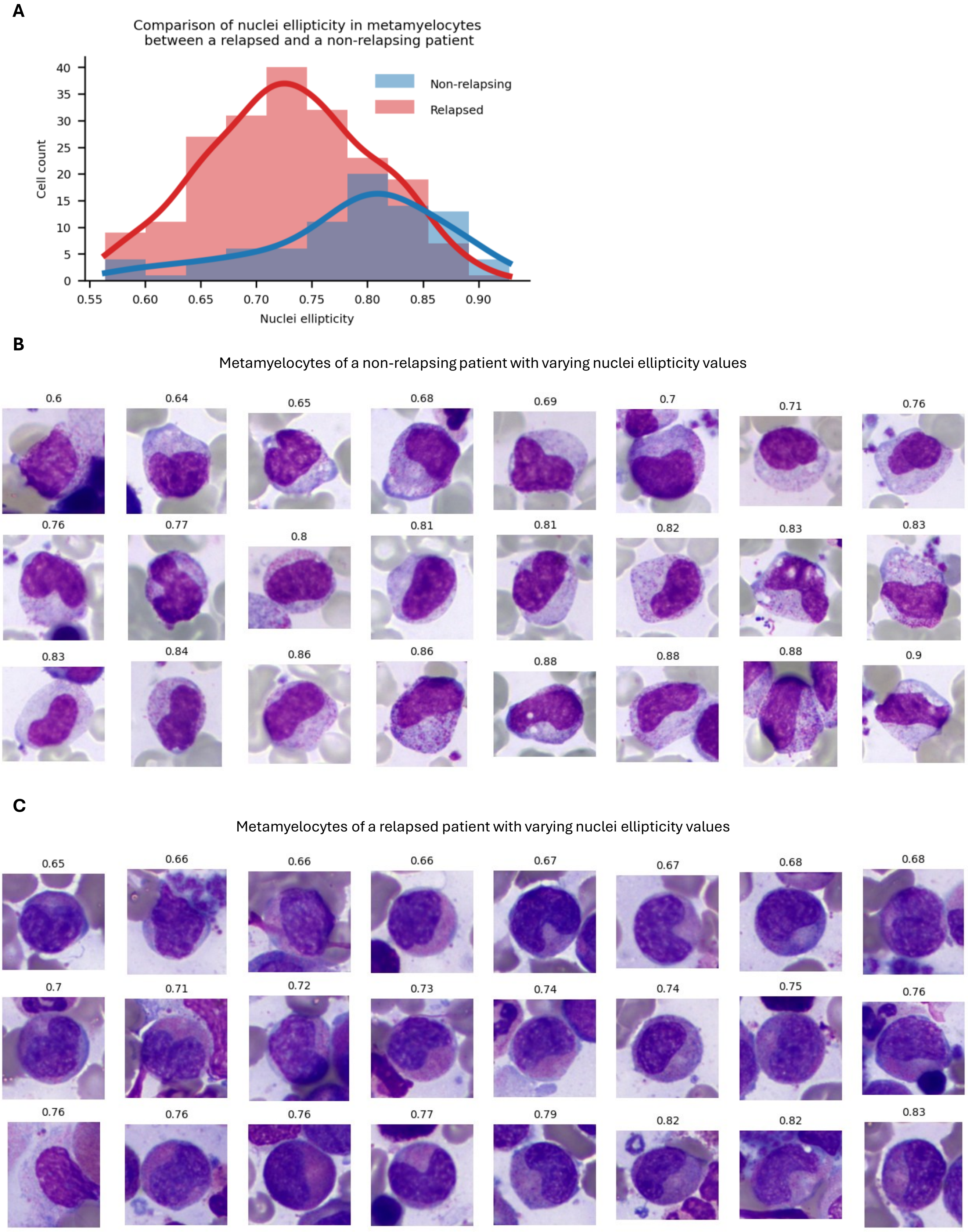

Supplementary Figure 3. Comparison of metamyelocytes based on nuclei ellipticity. (A) Distribution of metamyelocyte nuclei ellipticity values of a relapsed and a non-relapsed patient. (B) Metamyelocyte images from a non-relapsing and (C) a relapsed patient with varying nuclei ellipticity values.

A

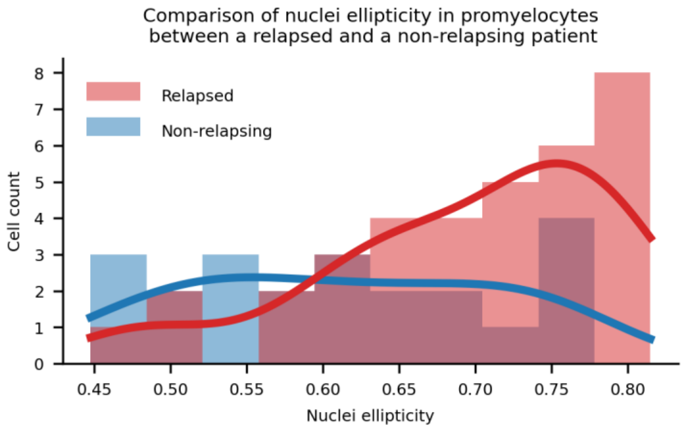

B

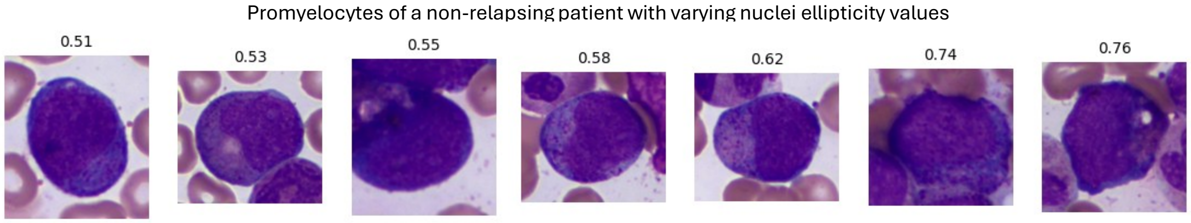

C

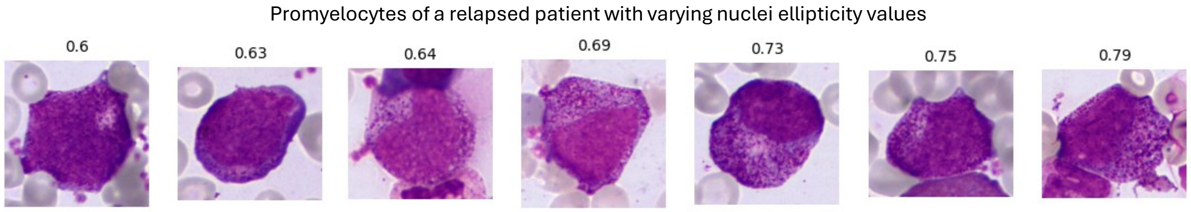

Supplementary Figure 4. Comparison of promyelocytes based on nuclei ellipticity. (A) Distribution of promyelocyte nuclei ellipticity values of a relapsed and a non-relapsed patient. (B) Promyelocyte images from a non-relapsing and (C) a relapsed patient with varying nuclei ellipticity values.

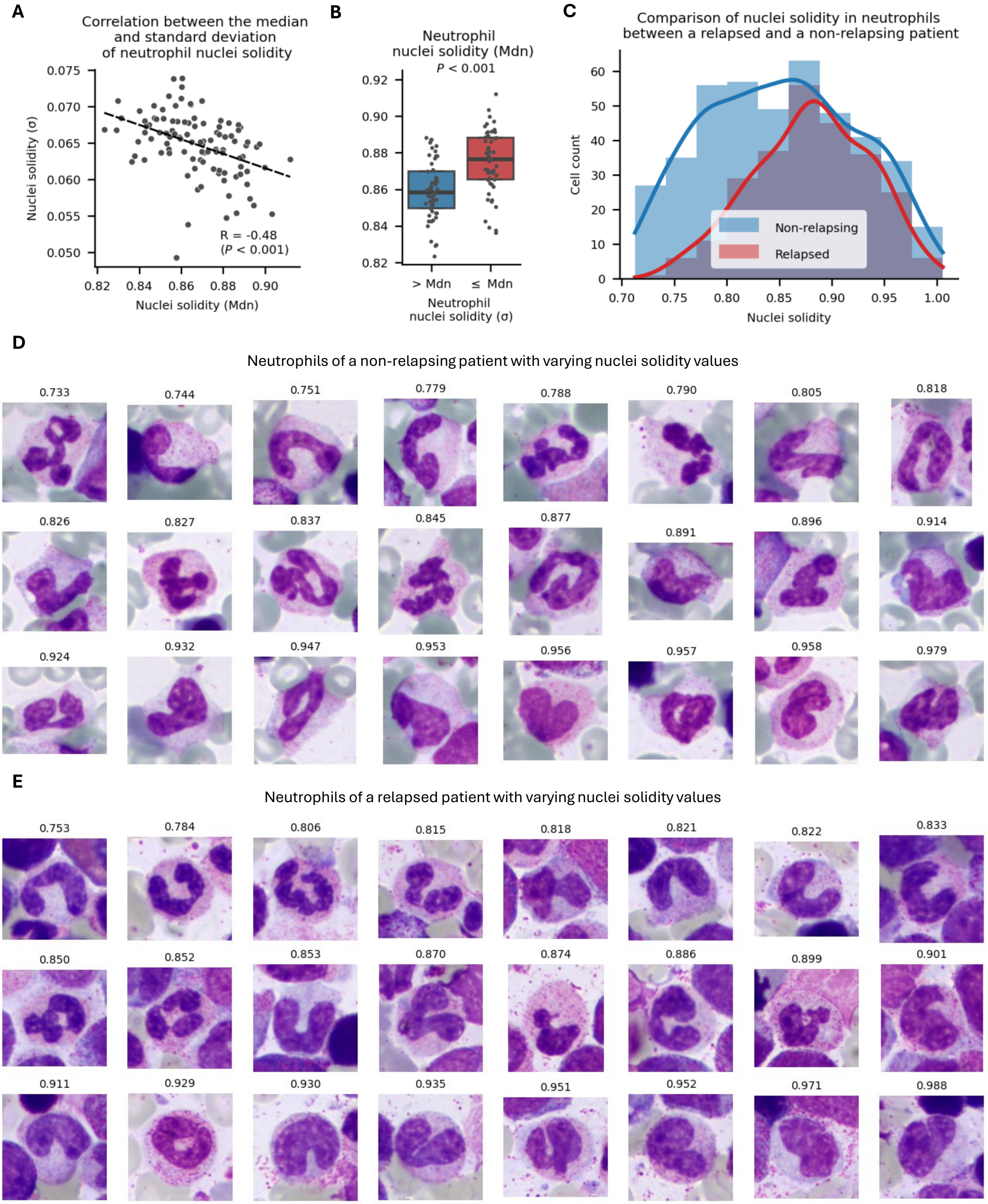

Supplementary Figure 5. Comparison of neutrophils based on nuclei solidity. (A) Scatter plot showing the relationship between standard deviation and median of neutrophil nuclei solidity, with correlation coefficient (R) and *P*-value shown (Spearman's rank correlation). (B) Box plot of the median nuclei solidity of neutrophils, categorized by the median value of neutrophil nuclei solidity variation (standard deviation), with *P*-value shown (Wilcoxon rank-sum test). (C) Distribution of neutrophil nuclei solidity values of a relapsed and a non-relapsed patient. (D) Neutrophil images from a non-relapsing patient and a (E) relapsed patient with varying nuclei solidity values. Abbreviations: σ, Standard deviation; Mdn, Median.

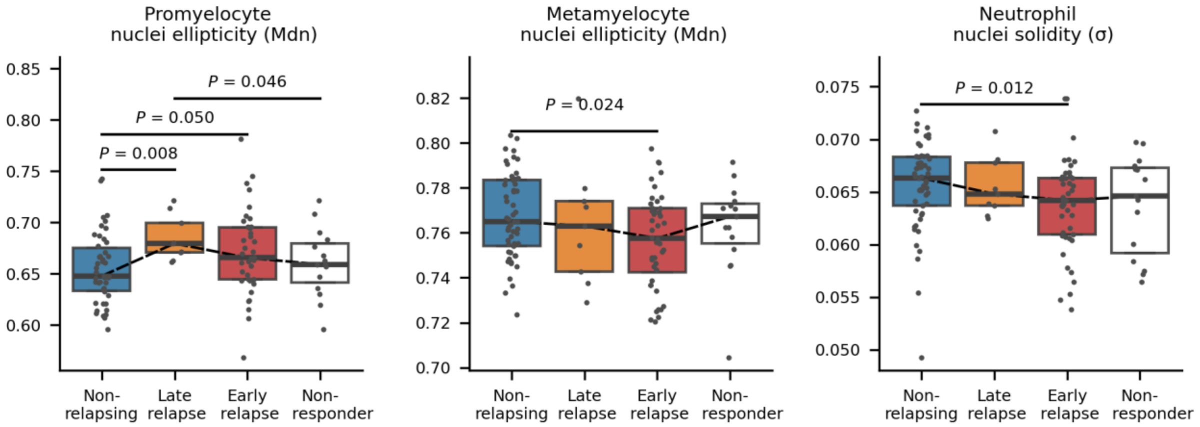

Supplementary Figure 6. Comparison of cell morphometry between chronic myeloid leukemia (CML) patients. The analysis included four CML patient groups: patients maintaining treatment-free remission (TFR) at 36 months, patients with a late (> 6, ≤ 36 months) or early relapse (≤ 6 months) following tyrosine kinase inhibitor (TKI) discontinuation and patients failing to respond to TKI during the first year after diagnosis according to European LeukemiaNet 2020 criteria. Box plots with individual data points for each morphometry variable, categorized by patient groups, with *P*-values shown (Wilcoxon rank-sum test). The black dashed line connects the groups by their medians. Abbreviations: σ, Standard deviation; Mdn, Median.

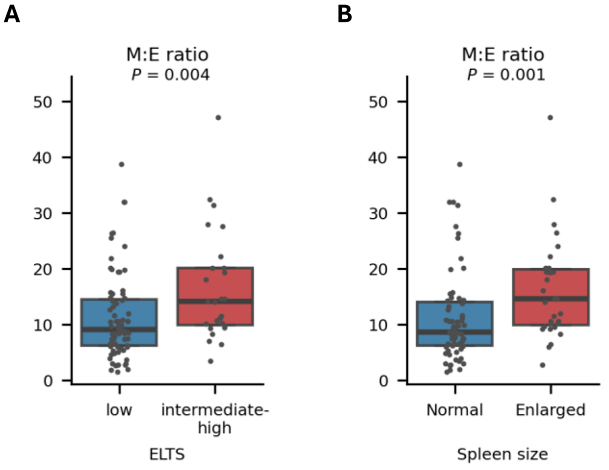

Supplementary Figure 7. Association of M:E Ratio and prognostic variables in chronic myeloid leukemia (CML). (A) Box plot of M:E ratio, categorized by EUTOS long-term survival (ELTS) risk class and (B) box plot of M:E ratio, categorized by spleen size, with *P*-values shown (Wilcoxon rank-sum test). Abbreviations: M:E Ratio, Myeloid to erythroid ratio where myeloid cells included promyelocytes, myelocytes, metamyelocytes, neutrophils, eosinophils, basophils, promonocytes, and monocytes. Erythroid cells included proerythroblasts and erythroblasts.

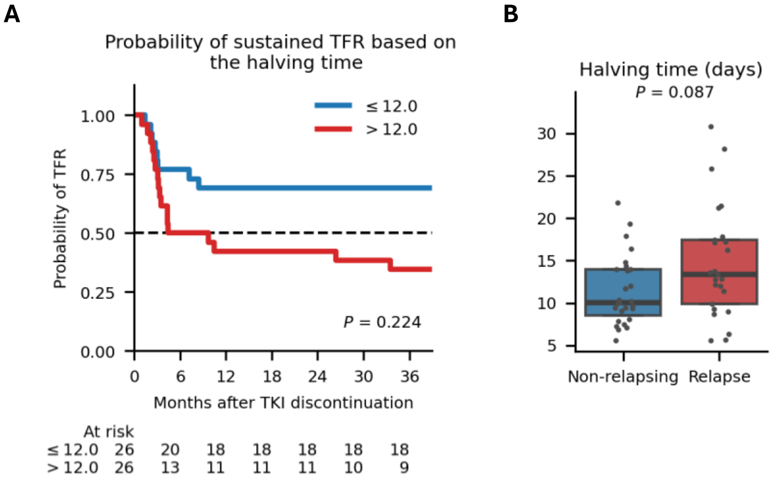

Supplementary Figure 8. Association of *BCR::ABL1* halving time with treatment-free remission (TFR) at 29 months after tyrosine kinase inhibitor (TKI) discontinuation. (A) Kaplan-Meier plot showing the probability TFR according to the halving time. Risk groups were defined based on the optimal cut-off halving-time value maximizing log-rank statistics: A blue line represent lower risk of relapse, and a red line indicate higher risk. Statistical comparisons were performed using the log-rank test, with adjusted *P*-value shown. A dashed horizontal line indicates the 50% probability of TFR. (B) Box plot the halving time, categorized by TFR maintenance, with *P*-value shown (Wilcoxon rank-sum test).

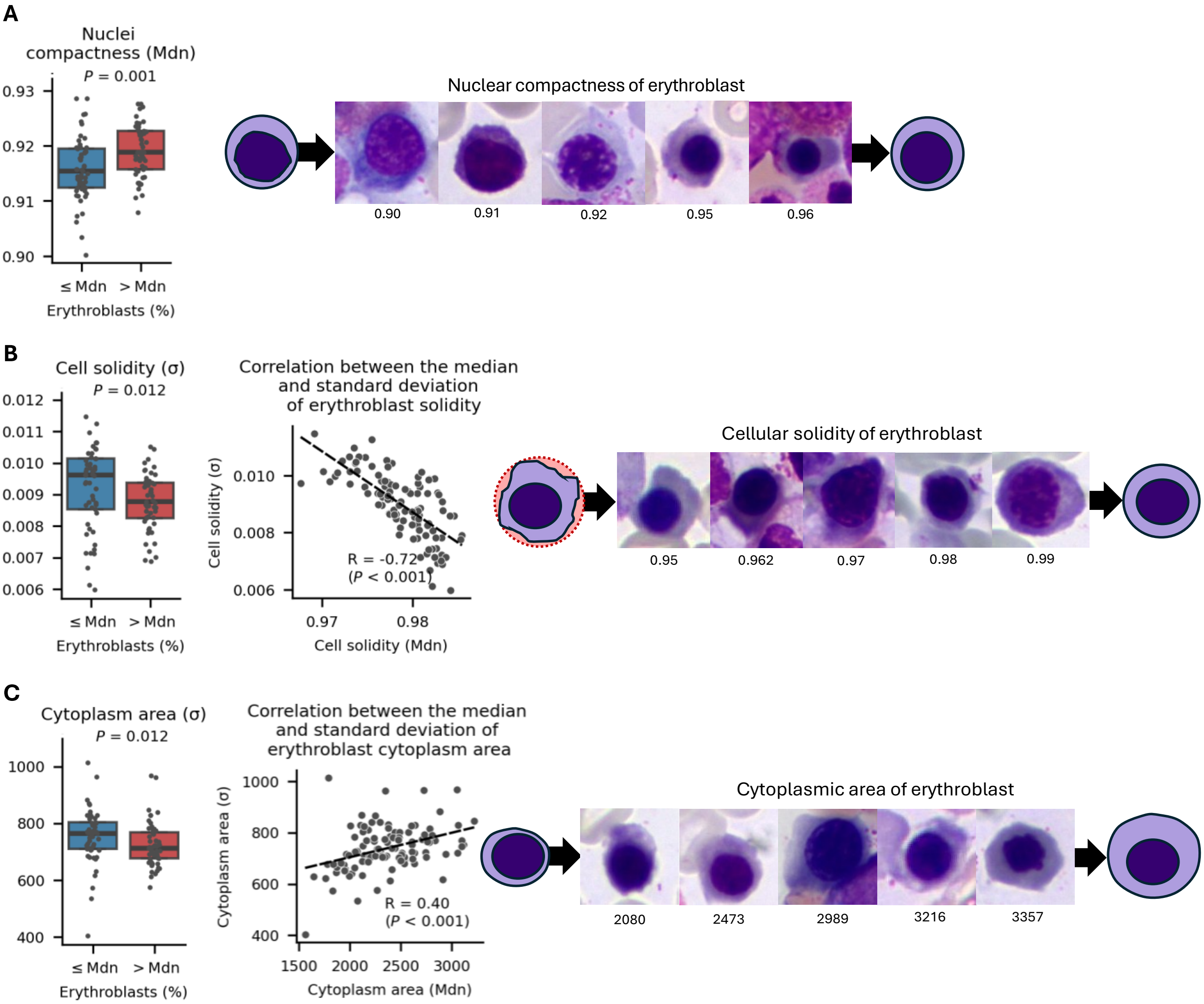

Supplementary Figure 9. Association between erythroblast proportion and morphology. (A) Box plot of median nuclear compactness categorized by the median erythroblast proportion. Illustration of erythroblasts with varying values of nuclear compactness. Compactness measures the ratio of the area to circle with the same perimeter. A maximum value of 1 represents a perfect circle whereas lower values indicate more irregular shape. (B) Box plot of the standard deviation of erythroblast solidity categorized by the median erythroblast proportion. Scatter plot showing the relationship between the median and standard deviation of erythroblast solidity. Illustration of erythroblasts with varying solidity values. Solidity measures the ratio of an object's area to its convex area. (C) Box plot of the standard deviation of erythroblast cytoplasmic area categorized by the median erythroblast proportion. Scatter plot showing the relationship between standard deviation and median cytoplasmic area. Illustration of erythroblasts with varying values of cytoplasmic area. Wilcoxon-rank sum test was used to compare two groups (*P*-values shown). Spearman's rank correlation was used to compare two continuous variables (correlation coefficient (R) and *P*-value shown). Abbreviations: σ, Standard deviation; Mdn, Median.

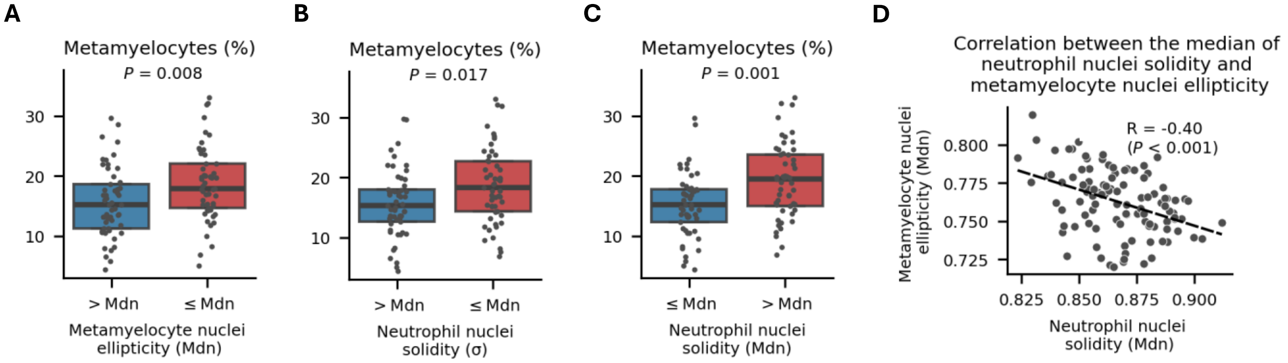

Supplementary Figure 10. Association between metamyelocyte proportion and morphology of metamyelocytes and neutrophils. (A) Box plot of metamyelocyte proportion, categorized by the median value of median metamyelocyte nuclei ellipticity. (B) Box plot of metamyelocyte proportion, categorized by the median value of neutrophil nuclei solidity variation (standard deviation). (C) Box plot of metamyelocyte proportion, categorized by the median value of median neutrophil nuclei solidity. Wilcoxon-rank sum test was used to compare two groups (*P*-values shown). (D) Scatter plot showing the relationship between the median neutrophil nuclei solidity and median metamyelocyte nuclei ellipticity, with correlation coefficient (R) and *P*-value shown (Spearman's rank correlation). Abbreviations: σ, Standard deviation; Mdn, Median.
